# Traditional medicine: a rare cause of lead poisoning in Western countries

**DOI:** 10.12688/f1000research.2-250.v1

**Published:** 2013-11-19

**Authors:** Halima Muller, Simon Regard, Nicole Petriccioli, Omar Kherad

**Affiliations:** 1 Service de Médecine Interne, Hôpital de la Tour, Geneva, Switzerland

## Abstract

A 42-year-old man from Bhutan was admitted to the emergency department with a 5-day history of abdominal pain, nausea and vomiting. Enhanced abdominal CT scan was found negative, however laboratory tests showed hemolytic anemia and basophilic stippling which are often seen in lead and heavy metal poisoning. Additional tests revealed a high level of lead in blood and urine. The patient was administered a chelator treatment with rapid improvement of the symptoms. A detailed interview revealed that the patient had been taking daily Bhutanese traditional medicines to treat a Bell’s palsy from which he had been suffering for a few months. The analysis of these medicines confirmed the presence of a high level of lead.

## Introduction

Lead poisoning is a multisystemic organ disease which can present with non-specific symptoms such as fatigue, anorexia, arthralgia, myalgia, neurological disorders, anemia abdominal pain and encephalopathy.

Basophilic stippling, which refers to the presence of blue granules of various sizes dispersed throughout the cytoplasm of the red blood cell may be seen in blood smears. Burton line, and nephropathy (Fanconi-type syndrome), are also described ^1^. The diagnosis of lead poisoning is based on clinician consideration to assess blood lead level and erythrocitic protoporphyrins. The treatment consists of the elimination of the source of exposure and a chelation therapy with DMSA (dimercaptosuccinic acid) or CaEDTA ^2, 3^. Timely diagnosis and identification of the source of exposure are critical in preventing the long-term consequences of lead poisoning.

Lead exposure and poisoning is still a major health issue in developing countries ^4^. It was estimated to be responsible for 8,955,000 disability-adjusted life years (DALYs) in 2004, i.e. 0.6% of all DALYs ^5^. Lead poisoning is most commonly caused by occupational exposure (especially in adults) ^6^. Other sources of lead poisoning include exposure to paint (especially in children), water and soil (especially in urban areas), food (including game) and toys (especially in children where toys may have lead-based paints), as well as complementary and alternative medicines (CAM). As a consequence of globalization, lead poisoning cases caused by CAM are increasingly reported in developed countries ^7^.

## Case presentation

A 42-year-old man from Bhutan with a history of hypertension was admitted on 26 ^th^ October 2011 to the emergency department of a secondary hospital in Geneva, Switzerland, complaining of epigastric pain with nausea and vomiting over the previous 5 days. A gastroscopy showed esophagitis and the patient was discharged with a proton pump inhibitor treatment. He consulted two days later complaining of persistent symptoms. Examination revealed pain on palpation of the right upper abdominal quadrant with no sign of peritonism.

The patient resides in Geneva and frequently travels to his home country. His occupation involves no risk of lead exposure.

Blood tests revealed a cholestatic liver abnormality and a hemolytic anemia with the hemoglobin level at 90 g/l. An abdominal ultrasound and a thoraco-abdominal CT scan proved normal. Serologies for HIV, cytomegalovirus, Epstein-Barr virus and hepatitis A, B, C were negative. A blood smear was performed and showed basophilic stippling that was highly evocative of a heavy metal poisoning. Blood and urinary lead levels were high at 80.8 mcg/dl and 208.8 mcg/g of creatinine, respectively. An increase in the urinary coproporphyrin III level up to 155.9 nmol/mmol (n<150nmol/mmol) was also noted.

**Figure 1.**
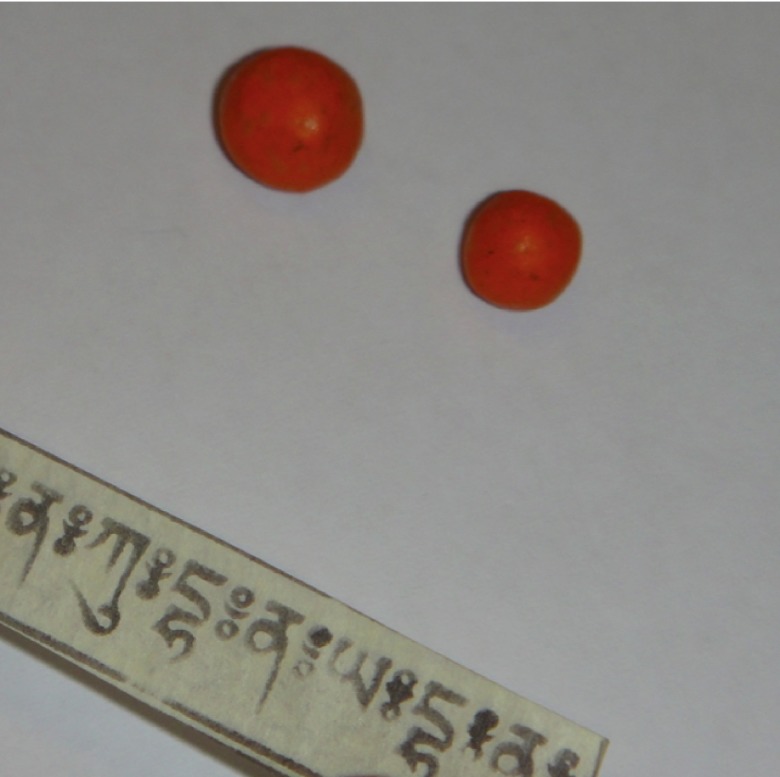
The analysis of the two pellets (0.5–1 cm Ø) showed the presence of a high level of lead on the surrounding red paint.

The origin of the intoxication was discovered to be due to the patient taking Bhutanese traditional medicines to treat a resolutive Bell’s palsy a few months earlier. These medicines were composed of parchment with ink writing and pellets ( Figure 1). The patient thought the drug comprised hair of a deceased local priest with therapeutic virtues. The quantitative analysis performed at the University Centre of Legal Medicine (CURML) showed the presence of high level of lead on the red paint surrounding the pellets (1.4 mg in each pellet) and a negligible level in the parchment with ink writing (<1ug).

The patient was administered an oral chelation therapy with DMSA (dimercaptosuccinic acid) 10 mg/kg 3 times daily for 5 days, and then 10 mg/kg 2 times daily for 14 days.

The symptoms rapidly improved and the patient was discharged 5 days later from hospital with outpatient control by his general physician. He was advised to stop taking the poisonous medicines. The case was reported to the Bhutanese health authorities for them to address the issue locally and implement an appropriate health care response.

## Discussion

Complementary and alternative medicines have become more frequent causes of lead poisoning in the past few decades. In 2007, a review article ^7^ found 76 case reports dating from 1966 to 2007 that involved traditional medicines from the Middle East, South America and India (Ayurveda). It is estimated that at least 15% of ingested lead is absorbed ^7^. Three main reasons explain the presence of lead in CAM medicines: involuntary contamination (e.g. soil where CAM plants grow), voluntary use of lead for therapeutic purposes and voluntary use of lead to increase the weight of medicines (the heavier the better) ^7^.

As a consequence of general globalization in medicine, CAM medicines are being increasingly used in Western countries as they become more popular and easily available on the internet ^8, 9, 10^.

CAM medicines play a very important though underrated role in developing countries where they are more accessible and affordable than pharmaceutical drugs and where they are consequently estimated to be used by between 66–80% of the population ^11^. It must be noted that CAM encompass a broad range of practices with various degrees of popular recognition. In our case, it appeared that the ingested balls were not part of any of the officially recognized practices included in the Bhutanese traditional medicine known as gSo-Ba Rig-Pa ^12^, hence making resolution of the issue more complex. Patients often do not overtly disclose their consumption of CAM drugs for fear of lack of understanding by their doctor ^13^. As a consequence the use and misuse of CAM medicines is certainly underreported.

Despite the body of literature on this topic, lead poisoning through CAM consumption remains a public health problem. Enhancing public awareness about the potential risks of many CAM approaches including exposure to harmful contaminants is a major objective of preventative health care. Meanwhile, it remains the responsibility of the physician to obtain a detailed history of medication use, including in developed countries where lead exposure and poisoning can also be reported.

## Consent

Written informed consent for publication of clinical details was obtained from the patient.
