# Supplementary material for: Traditional medicine: a rare cause of lead poisoning in Western countries
Source: F1000Res. 2013 Nov 19;2:250. [Version 1] doi: 10.12688/f1000research.2-250.v1 (PMC3869481; doi:10.12688/f1000research.2-250.v1)
Supplement: Peer review history

# Peer review: Referee response for version 1

**Published:** 2013-11-28

**Authors:** Bruno Mégarbane, Robert Garnier

**Affiliations:** 1 Department of Medical and Toxicological Critical Care, Lariboisière Hospital, Paris-Diderot University, Paris, France; 2 Fernand Widal Hospital

The authors report an interesting case of lead poisoning following the administration of traditional remedies. This is a rather well-known situation, with several published cases, mainly occurring in the developing countries where traditional medicines are widely used. The case is well-written and discussed.

I have only minor comments:

1. Severe lead poisoning is responsible for abdominal pain which may be associated with nausea and/or vomiting but is generally preceded and associated with constipation. Was this patient constipated?
2. Hemolysis and cytolytic (rather than cholestatic) hepatitis are manifestations of subacute (or acute) lead poisoning; could the authors comment on their finding?
3. The clinical (and biological) course of this lead poisoning case could be better described, regarding the onset of renal complications, course of hemolysis and hepatitis, as well as blood lead concentration one or two weeks after chelation.
4. Arthralgia and myalgia are rare features of lead poisoning, as written in the introduction section. The main implications of lead poisoning are neurological (encephalopathy and peripheral neuropathy), hematological and renal (tubular nephropathy, then tubulo-intersitial and glomerular nephropathy).
5. In adults, digestive absorption of lead is poorer (5-10 %) than indicated in the discussion section.

We have read this submission. We believe that we have an appropriate level of expertise to confirm that it is of an acceptable scientific standard.

# Peer review: Referee response for version 1

**Published:** 2013-11-25

**Authors:** Arnaud Perrier

**Affiliations:** 1 Department of Internal Medicine, University Hopitals of Geneva (HUG), Geneva, Switzerland

The authors report an interesting case of lead poisoning by a Bhutanese alternative medicine treatment. The interest resides in the frequency of complementary and alternative medicine (CAM) as a source of accidental lead poisoning, a fact most physicians are not aware of.  Also, the conclusion that CAM medications should be part of treatment history-taking is important and sound advice.

Minor points to be improved:

1. The reference interval for blood and urine lead level should be provided.
2. It would be interesting to know how the follow-up of such a case should be performed. At what intervals should lead measurements be repeated?

I have read this submission. I believe that I have an appropriate level of expertise to confirm that it is of an acceptable scientific standard.
